# Neoadjuvant Four-Drug Combination Therapy for NSCLC With EGFR Mutation Avoiding Total Pneumonectomy

**DOI:** 10.3389/fonc.2020.01145

**Published:** 2020-07-16

**Authors:** Jingpei Li, Ke Xu, Weipeng Cai, Yalei Zhang, Xin Zeng, Fei Cui, Zhexue Hao, Jun Liu, Jianxing He

**Affiliations:** ^1^Department of Thoracic Surgery, The First Affiliated Hospital of Guangzhou Medical University, Guangzhou, China; ^2^Guangzhou Institute of Respiratory Health (GIRH), Guangzhou, China; ^3^State Key Laboratory of Respiratory Disease, National Clinical Research Center for Respiratory Diseases, Guangzhou, China; ^4^Department of Pathology, The First Affiliated Hospital of Guangzhou Medical University, Guangzhou, China

**Keywords:** neoadjuvant, EGFR mutation, targeted (selective) treatment, chemothearpy, surgery

## Abstract

We report a case of successful neoadjuvant four-drug combination therapy to avoid total pneumonectomy. A 33-year-old male patient was diagnosed with locally advanced non-squamous NSCLC harboring EGFR mutation in the left lower lobe. The patient experienced significant clinical downstaging after two cycles of neoadjuvant therapy, including icotinib, carboplatin, pemetrexed, and bevacizumab. He underwent a successful lobectomy avoiding pneumonectomy. The patient showed no recurrence in the follow-up of a chest computed tomographic scan, which is 17 months after surgery. The promising results of this neoadjuvant combination therapy provided a novel therapeutic option for patients with locally advanced EGFR-mutated NSCLC facing total pneumonectomy.

## Introduction

Even with multidisciplinary treatment, the 5-year overall survival rates in stage III NSCLC patients are ~25 to 35% (1). Molecularly targeted agents have been shown to improve the overall survival for patients with NSCLC harboring epidermal growth factor receptor (EGFR) mutation in the setting of advanced disease. Researchers have begun to seek the possibility of applying a single agent of EGFR- tyrosine kinase inhibitor (TKI) from advanced to earlier stage (2). However, despite achieving significant downstaging of tumors, local or distant disease relapsed in less than 1 year. Various combination therapy achieved the promising results in the setting of advanced stage, including chemotherapy/EGFR-TKI (3, 4), chemotherapy/Bevacizumab (5), EGFR-TKI/Bevacizumab (6, 7), and eventually chemotherapy/EGFR-TKI/Bevacizumab (8). Currently, some prospective trials (9–11) had investigated the critical role of EGFR-TKI (gefitinib or erlotinib) in neoadjuvant therapy, the objective response rate (ORR) was ranged from 42.1 to 54.5%, and the rate of major pathological regression (MPR) was from 9.7% up to 24.2%. Considering the neoadjuvant EGFR-TKI combined with chemotherapy or anti-angiogenesis was tolerable, and could improve the radical resection rate, so we proposed a four-agent combination therapy for locally advanced non-squamous non-small-cell lung cancer harboring EGFR mutations.

## Case Description

**Figure 1:**
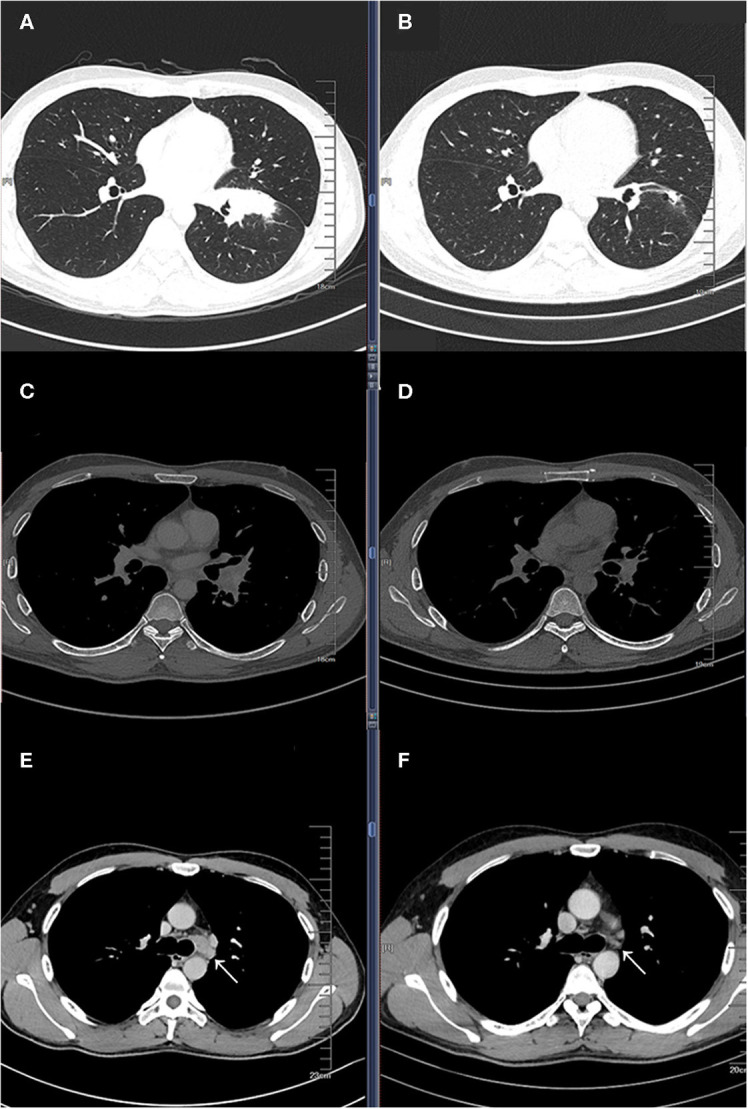
Baseline computed tomography (CT) showing a lung mass in the left lower lobe pulmonary window **(A)** and mediastinal window **(C)**, and an enlarged left lower paratracheal (4L) lymph node **(E)**; CT images after two cycles of four drug combination therapy showing partial response in the pulmonary window **(B)** and mediastinal window **(D)**, and a significantly reduction in the 4L lymph node **(F)**.

A 33-year-old male smoker presented with cough and sputum was admitted to the department of thoracic surgery. Computed tomography (CT) revealed a lung mass in the left lower lobe (Figures 1A,C) and multiple hilar and mediastinal lymph nodes (Figure 1E). Cerebral magnetic resonance imaging and the bone scan did not show any lesion. The bronchoscopic biopsy confirmed the diagnosis of lung adenocarcinoma (cT2bN2M0, stage IIIA), and amplification refractory mutation system (ARMS)-polymerase chain reaction (PCR) showed an EGFR 19 exon deletion mutation. Multidisciplinary team discussion suggested neoadjuvant therapy over total pneumonectomy. Based on our previous experience of various combination therapies within our institute, four-drug combination therapy with fixed regimens was proposed, including icotinib 125 mg po, tid, and intravenous carboplatin 150 mg D1-D2, pemetrexed 800 mg D1, and bevacizumab 300 mg D1, given a young age and good general status. After two cycles of neoadjuvant therapy, the Positron emission tomography/Computed tomography (PET/CT) revealed that the tumor was reduced from 4.6 × 2.0 cm to 1.4 × 1.1 cm (Figures 1B,D) with a normal standard uptake value (SUV), and there were no enlarged mediastinal or hilar lymph nodes (Figure 1F). No grade 3/4 adverse event (AE), including hemoptysis, was experienced, while rash and gastrointestinal symptoms were the most frequent AEs based on the patient's self-report.

**Figure 2:**
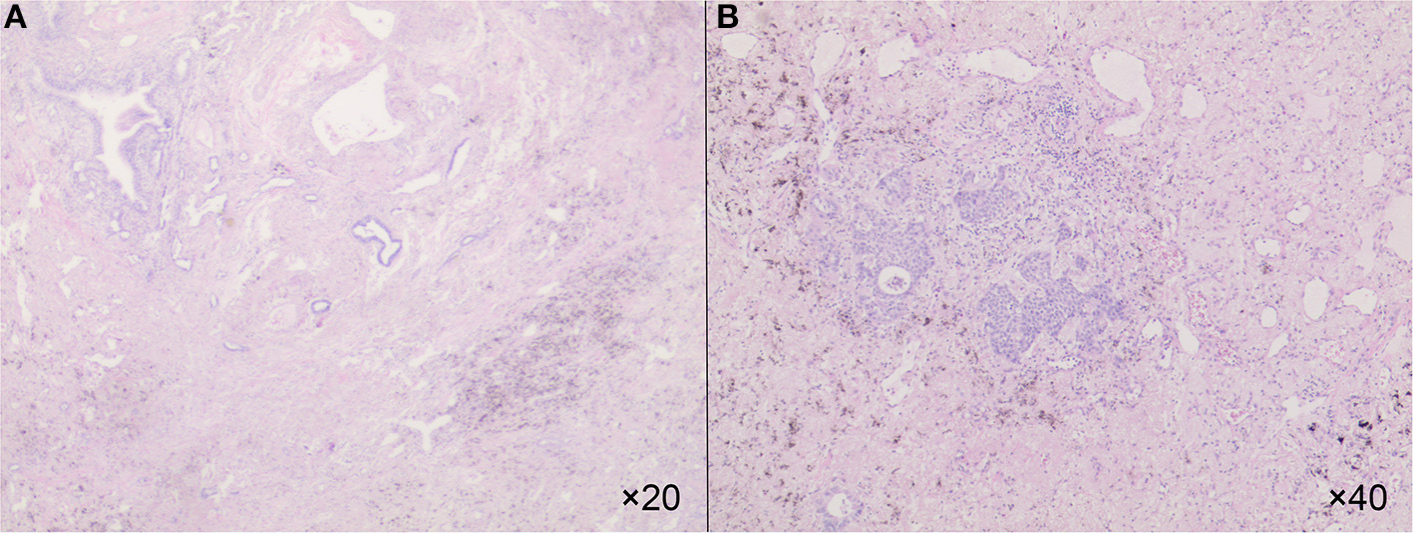
The HE showed an increase in the number of residue cancer cells, but fibrosis still predominated in 20× **(A)** and in 40× **(B)**.

**Figure 3:**
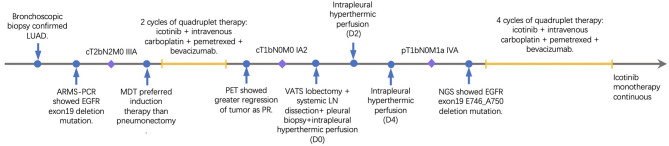
The timeline showing the relevant data of treatment.

The re-evaluation workup suggested a significant downstaging of the tumor (cT1bN0M0, stage IA2). Then 6 weeks after the last cycle of combination regimens were given, a left lower lobectomy was proposed. However, an occult parietal pleural metastasis was detected during surgery. In the operative fields, we observed tissue edema and hyperemia of the lung and moderate fibrosis of the external coat of vessels. The patient underwent a video-assisted thoracoscopic surgery (VATS) left lower lobectomy, systematic lymphadenectomy, pleural biopsy, and intrapleural hyperthermic perfusions, as well as two additional intrapleural hyperthermic perfusion performed on the next 2 days. The estimated intraoperative blood loss was nearly 200 ml due to mild tissue hyperemia of the lung. The perioperative recovery was uneventful. The patient was discharged on the seventh postoperative day. Pathology confirmed the diagnosis of lung adenocarcinoma with pleural metastasis (ypT1bN0M1a, stage IVA). The histologic findings indicated significant tumor regression. It was characterized by an increase in the number of residue cancer cells, but fibrosis still predominated (Figures 2A,B). No lymph nodes metastasis was found at the dissected nodes station 5 (0/1), 7 (0/2), and 10 (0/1); no vascular cancer thrombus and no residual tumor in the bronchial stumps were observed. The next-generation sequencing (NGS) of pathological specimens also confirmed EGFR 19 exon E746_A750 deletion mutation. Thus, after 1 month for recovery postoperatively, an additional of four-drug combination therapy was successfully conducted every 3 weeks ± 3 day for four cycles. Single-agent icotinib was continued, with a follow-up chest CT scan every 3 months and PET/CT every 6 months. The latest follow-up recorded no disease progression in a chest CT scan when it was 16.7 months after surgery and 19.4 months after initial treatment. The timeline of treatment with relevant data of care was shown in Figure 3.

## Discussion

The patient's course illustrates the successful use of four-drug combination therapy to treat a locally advanced non-squamous NSCLC harboring EGFR mutation. The patient experienced significant tumor downstaging and underwent lobectomy instead of total pneumonectomy.

Patients with locally advanced NSCLC, especially N2 disease, should be considered for neoadjuvant chemotherapy or chemoradiotherapy based on NCCN guidelines. Although EGFR-TKI monotherapy is considered the standard first-line treatment for patients with TKI-sensitive EGFR mutations, most patients develop recurrence within ~1 year. The rationale for concurrent use of cytotoxic agents and bevacizumab is to reduce or even avoid potential *de novo* resistance (12). Sugawara (13) obtained the median progression-free survival (PFS) 18.3 and 15.3 months for the concurrent and sequential alternating regimens with gefitinib and carboplatin/pemetrexed in patients with EGFR-mutated NSCLC in a randomized phase II study, which is more promising in comparison to the PFS in previous studies with first-line gefitinib monotherapy (14). Two randomized clinical trials demonstrated erlotinib combined with bevacizumab harvest a median PFS of 16.0–16.9 months, which was significantly better than erlotinib monotherapy (7).

Under the expectation to provide high-level, customized treatment for patients with resectable NSCLC, several phase II clinical trials had revealed the efficacy and safety on respect of preoperative TKI therapy (10, 11). EMERGING-CTONG 1103 showed the improved ORR for neoadjuvant erlotinib with 54.1% compared to gemcitabine plus cisplatin chemotherapy with 34.3% and doubling PFS with erlotinib (21.5 months) vs. GC chemotherapy (11.4 months; *p* < 0.001). Zhang's study showed similar ORR to the previous study, and patients with MPR were associated with improved survival while no patients reported grade 3 or 4 AEs. These results suggest that biomarker-guided neoadjuvant EGFR-TKI treatment in resectable NSCLC is promising. Hence, we combined these four drugs to optimize the treatment effect by avoiding potential *de novo* resistance. This case did reflect a promising effect. It is possible that TKI played a key role in the significant response of preoperative therapy. However, the first generation of EGFR-TKI is reversible TKI, which leaves it a tumor inhibitor. Chemotherapy provides a curative potential, which is critical for significant response of preoperative therapy and long-term tumor control. Since no neoadjuvant therapy can guarantee a full transfer to complete resection. In the long-term, however, bevacizumab may reduce potential *de novo* resistance if complete resection is unachievable. So it was important to include bevacizumab in the combination therapy.

Apart from a slight increase in intraoperative blood loss, there were no significant adverse events reported in this case, which was mainly the result of careful case selection and a fixed lower dosage of the cytotoxic agents and is far from a standard requirement. In our institute, we have attempt to apply the EGFR-TKI, EGFR-TKI/Chemotherapy, and Chemotherapy/Bevacizumab in several locally advanced NSCLC patients. The regimens of Chemotherapy were all adjusted to this fixed lower dosage. No significant adverse event was observed in the treatment period. All patients underwent surgery successfully. Yet, during the surgery, different degrees of tissue edema and hyperemia of the lung and fibrosis of the external coat of vessels in the operative fields. In general, any therapy involving EGFR-TKI was expecting moderate to severe fibrosis of the external coat of vessels. There are a few reasons that accounted for the choice of icotinib in this patient. First, we did not choose erlotinib or gefitinib because there was a beneficence project in China so that the patient can acquire a free prescription after 10 months of using icotinib. So it was more economical to use this drug, given the non-inferior to gefitinib in efficacy with favorable safety in non-selected or EGFR-mutant NSCLC patients (15). Second, we did not choose osimertinib because it would be 10-fold higher than icotinib and nearly 20 times of cost if insurance was taken into consideration. Though median progression-free survival was significantly longer with osimertinib than SoC EGFR-TKI (16), the overall survival of the FLAURA study of osimertinib was not reported until recently (17).

In conclusion, treatment with this neoadjuvant combination therapy provided a novel therapeutic option for patients with locally advanced EGFR-mutated NSCLC facing total pneumonectomy.

## Ethics Statement

The studies involving human participants were reviewed and approved by Ethics Committee of the First Affiliated Hospital of Guangzhou Medical University. Written informed consent was obtained from the individual(s) for the publication of any potentially identifiable images or data included in this article.

## Author Contributions

JL and JH was involved in planning and supervised the study. All authors wrote the manuscript and designed the figures. All authors contributed to the article and approved the submitted version.

## Conflict of Interest

The authors declare that the research was conducted in the absence of any commercial or financial relationships that could be construed as a potential conflict of interest.
