# Erratum: Neoadjuvant Four-Drug Combination Therapy for NSCLC With EGFR Mutation Avoiding Total Pneumonectomy

**DOI:** 10.3389/fonc.2020.01573

**Published:** 2020-07-29

**Authors:** Frontiers Production Office

**Affiliations:** Frontiers Media SA, Lausanne, Switzerland

**Keywords:** neoadjuvant, EGFR mutation, targeted (selective) treatment, chemothearpy, surgery

Due to an editorial error, the wrong version of the manuscript was typeset and published. The publisher apologizes for this mistake. The original article has been updated.

